# Heterochromatin and numeric chromosome evolution in Bignoniaceae, with emphasis on the Neotropical clade *Tabebuia* alliance

**DOI:** 10.1590/1678-4685-GMB-2018-0171

**Published:** 2020-02-27

**Authors:** Joel M.P. Cordeiro, Miriam Kaehler, Luiz Gustavo Souza, Leonardo P. Felix

**Affiliations:** 1 Universidade Federal da Paraíba, Centro de Ciências Agrárias, Departamento de Ciências Biológicas, Campus II, Areia, PB, Brazil.; 2 Mulleriana: Sociedade Fritz Müller de Ciências Naturais, Curitiba, PR, Brazil.; 3 Universidade Federal de Pernambuco, Centro de Ciências Biológicas, Departamento de Botânica, Recife, PE, Brazil.

**Keywords:** Chromosome number, CMA/DAPI, Handroanthus, polyploidy

## Abstract

Bignoniaceae is a diverse family composed of 840 species with Pantropical distribution. The chromosome number 2*n* = 40 is predominant in most species of the family, with *n* = 20 formerly being considered the haploid base number. We discuss here the haploid base number of Bignoniaceae and examine heterochromatin distributions revealed by CMA/DAPI fluorochromes in the *Tabebuia* alliance, as well as in some species of the Bignonieae, Tecomeae, and Jacarandeae tribes. When comparing the chromosome records and the phylogenies of Bignoniaceae it can be deduced that the base number of Bignoniaceae is probably *n* = 18, followed by an ascendant dysploidy (*n* = 18 → *n* = 20) in the most derived and diverse clades. The predominant heterochromatin banding patterns in the *Tabebuia* alliance were found to be two terminal CMA^+^ bands or two terminal and two proximal CMA^+^ bands. The banding pattern in the *Tabebuia* alliance clade was more variable than seen in Jacarandeae*,* but less variable than Bignonieae. Despite the intermediate level of variation observed, heterochromatin banding patterns offer a promising tool for distinguishing species, especially in the morphologically complex genus *Handroanthus*.

## Introduction

Bignoniaceae is a Pantropical family composed mostly of trees and lianas, and includes 82 genera and 840 species (Fischer *et al.*, 2004; Lohmann and Ulloa, 2016). Eight tribes are nested within the family: Bignonieae, Catalpeae, Coleeae, Crescentieae, Jacarandeae, Oroxyleae, Tecomeae, and Tourrettieae, plus the informal Crescentiina clade, that comprises the Neotropical and Palaeotropical subclades (Olmstead *et al.*, 2009). While the morphological features of most tribes of Bignoniaceae are well-characterized, the Crescentiina clade and its subclades are well-sustained lineages, although without clear morphological synapomorphies (Grose and Olmstead, 2007; Olmstead *et al.*, 2009). The Crescentiina clade comprises two informal lineages: the exclusively Neotropical *Tabebuia* alliance and the Paleotropical clade with Asian and African genera (Olmstead *et al.*, 2009). The *Tabebuia* alliance has 14 genera and 147 species of trees and shrubs that have composite and palmate leaves (Grose and Olmstead, 2007). Most species within that clade belong to *Tabebuia* Gomes ex DC. and *Handroanthus* Mattos, while the remaining genera are smaller but widely-distributed in the Americas (Gentry, 1992; Grose and Olmstead, 2007). There is great morphological variability within the *Tabebuia* alliance, so that the delimitation of its species is often difficult.[Bibr B54]

From a cytogenetic point of view, the Bignoniaceae family comprises two groups with distinct karyotypes. The first group has a wide range of chromosome numbers (2*n* = 22, 28, 30, 36, 38, 40 and 42) and includes the tribes Jacarandeae, Tecomeae, Oroxyleae, and the two genera *Argylia* D.Don and *Delostoma* D.Don (Moore, 1974; Goldblatt and Gentry, 1979; Piazzano, 1998; Piazzano *et al.*, 2015). The second group has the prevailing chromosome number 2*n* = 40, and includes Bignonieae, Catalpeae, and the Crescentiina clade (Goldblatt and Gentry, 1979; Piazzano, 1998; Alcorcés de Guerra, 2002; Ortolani *et al.*, 2008; Firetti-Leggieri *et al.*, 2011; Piazzano *et al.*, 2015; Cordeiro *et al.*, 2016a, 2017). Ploidy variations (2*n* = 60, 80 and 120) were found for a few species of the tribe Bignonieae and the clade *Tabebuia* alliance from the second group (Piazzano, 1998; Alves *et al.*, 2013; Piazzano *et al.*, 2015; Cordeiro *et al.*, 2017).

Most species of Bignoniaceae show *n* = 20, and it has been proposed that *x* = 20 is the haploid base number for the family (Goldblatt and Gentry, 1979; Piazzano, 1998; Piazzano *et al.*, 2015). However, when confronting the known chromosome numbers of Bignoniaceae and the phylogenetic analyses of Olmstead *et al.* (2009), it became evident that the most primitive clades (such as Jacarandeae) are *x* = 18, suggesting that a different number from 20 could be the haploid base number of the family.

Chromosome numbers and morphologies are the features most used in karyotype analyses and ground cytotaxonomy studies (Guerra, 2008), although those characters can be uninformative in groups where chromosome numbers are stable and the chromosomes are small (< 3 μm) (Guerra, 2000, 2012). Bignoniaceae have chromosome sizes of ~2 μm, meta- submetacentric morphology, and 2*n* = 36 or 40 is predominant in the majority of species (Goldblatt and Gentry, 1979; Piazzano *et al.*, 2015; Cordeiro *et al.*, 2016b, 2017). Banding pattern characterizations can therefore often help discriminate between cytotypes with stable chromosome numbers, sizes and morphologies. The fluorochromes Chromomycin A_3_ (CMA) and 4’6-diamidino-2-phenylindole (DAPI) are specific for GC-rich (CMA) or AT-rich (DAPI) regions respectively, and usually stain regions with tandem repeats of non-coding DNA (Schweizer, 1976; Guerra, 2000). They have been used mainly to characterize karyotypes with chromosomes that have the same size and morphology, and to differentiate the karyotypes of species with identical chromosome numbers (see Almeida *et al.*, 2007; Barros e Silva *et al.*, 2010; Cordeiro *et al.*, 2016b; Almeida *et al.*, 2016). The different patterns found can help determine taxonomic distinctions and clarify relationships among species (Carvalho *et al.*, 2005; Almeida *et al.*, 2007; Oliveira *et al.*, 2015), as well as contribute to the description of new taxa, such as *Epidendrum sanchezii* E.Pessoa & L.P.Felix (Pessoa *et al.*, 2014), *Ameroglossum manoel-felixii* L.P.Felix & E.M.Almeida (Almeida *et al.*, 2016), and *Spondias bahiensis* P.Carvalho, Van den Berg and M.Machado (Almeida *et al.*, 2007; Machado *et al.*, 2015). Preliminary studies in the tribe Jacarandeae (Cordeiro *et al.*, 2016b) indicated that heterochromatin distribution appeared to follow a specific pattern (8-16 CMA^+^ terminal bands), while in the tribe Bignonieae (Cordeiro *et al.*, 2017) heterochromatin distribution is quite variable among the species. That result demonstrates that regions rich in GC base pairs (CMA^+^) can be variable even among closely related species of Bignoniaceae, and that a specific pattern for each group or tribe may not exist.

The main objective of this work was to describe the cytotaxonomic differences between related species of Bignoniaceae (mainly in the Neotropical lineage of the *Tabebuia* alliance clade) by examining their heterochromatin distributions, and discuss the haploid base number of the Bignoniaceae based on compilations of the chromosome numbers known for all lineages of the family.

## Materials and Methods

### Taxon sampling

**Figure 1:**
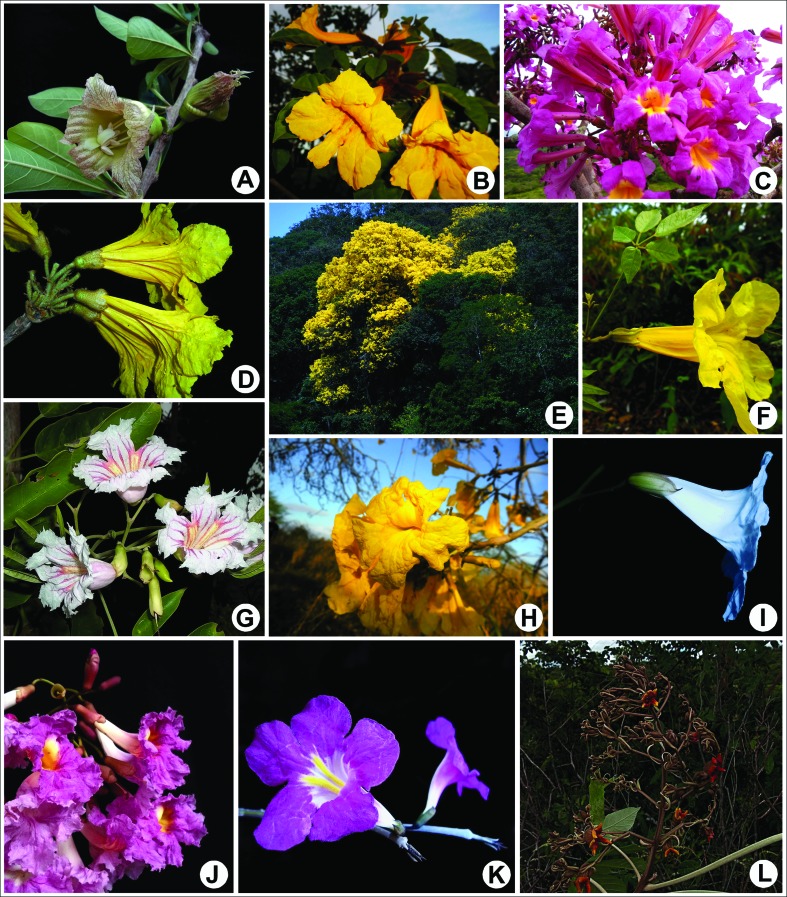
Some of the species of the Neotropical lineage *Tabebuia* alliance clade sampled. A. *Crescentia cujete*, B. *Handroanthus chrysotrichus*, C. *H. impetiginosus*, D. *H. ochraceus*, E. *H. serratifolius*, F. *H. umbellatus*, G. *Sparattosperma leucanthum*, H. *Tabebuia aurea*, I. *T. elliptica*, J. *T. rosea*, K. *T. roseoalba*, L. *Zeyheria tuberculosa*.

Heterochromatin banding patterns of 12 species of the *Tabebuia* alliance clade were analyzed (Figure 1), as well as those of three species of Jacarandeae, two species of Tecomeae, and two species of Bignonieae tribes. The species, vouchers, and primarily karyological information are presented in Table 1. The vouchers were deposited in the EAN herbarium. An average of three specimens of each species were grown in plastic pots in the experimental garden of the Centro de Ciências Agrárias of the Universidade Federal da Paraíba. When the roots reached 2 cm in length, 15 root tips per specimen were excised and analyzed.

**Table 1:** Species of Bignoniaceae analyzed and their main karyological parameters. Heterochromatin patterns: A - large telomeric CMA^+^ bands, B - small telomeric CMA^+^ bands, C - proximal CMA^+^ bands, F - lack of heterochromatic bands. Abbreviations of the Voucher: JMPC - Joel Maciel Pereira Cordeiro, LPF - Leonardo Pessoa Felix, EMA - Erton Mendonça de Almeida, SAAL - Saulo Antonio Alves de Lima. Abbreviations in the Origin: PB - Paraíba State, BA - Bahia State, PI - Piauí State, and MG - Minas Gerais State, Brazil.

Tribe/Alliance/species	Voucher	Origin	2*n*	Median size (μm)	Heterochromatin patterns	Figure
**Jacarandeae**						
*Jacaranda jasminoides* (Thunb.) Sandwith.	JMPC, 131	Sertãozinho-PB	36	2.09	6A + 4B + 26F	3A
*J. mimosifolia* D.Don	LPF, 14457	Areia-PB	36	1.84	6A + 2B + 28F	3B
*J. praetermissa* Sandwith^*^	LPF, 17606	Serra da Capivara-PI	36	2.19	2A + 8B + 26F	3C
**Tecomeae**						
*Podranea ricasoliana* (Tanfani) Sprague	JMPC, 135	Areia-PB	38	1.07	6B + 32F	3D
*Tecoma stans* (L.) Juss. ex Kunth	LPF, 14412	Paulo Afonso-BA	36	1.16	2A + 4C + 30F	3E
**Bignonieae**						
*Anemopaegma citrinum* Mart. ex DC.^**^	JMPC, 1254	Pico do Jabre-PB	40	1.32	2A + 2B + 2D + 34F	3F
*Fridericia chica* (Bonpl.) L.G.Lohmann^*^		Manaus, AM	40	1.76	6A + 26B + 6E + 2F	3G
*Tabebuia* alliance						
*Handroanthus chrysotrichus* (Mart. ex DC.)	EMA, 814	Campina Grande-PB	80	1.44	4A + 4B + 4C + 68F	1B, 3H
*H. impetiginosus* (Mart. ex DC.) Mattos	SAAL, 86	Areia-PB	40	1.39	2A + 2C + 36F	1C, 3I
*H. ochraceus* (Cham.) Mattos	SAAL, 84	João Pessoa-PB	80	1.42	6B + 74F	1D, 3J
*H. serratifolius* (Vahl.) S. O. Grose. Mattos	JMPC, 251	Areia-PB	120	1.63	4A + 6B + 4C + 106F	1E, 4A
*H. umbellatus* (Sond.) Mattos^*^	JMPC, 1043	Sertãozinho-PB	40	1.66	2A + 2B + 4C + 32F	1F, 4B
*Sparattosperma leucanthum* (Vell.) K.Schum.^*^	LPF, 15402	Alvorada de Minas-MG	40	1.55	2A + 38F	1G, 4C
*Tabebuia aurea* (Silva Manso) Benth. & Hook.f. ex S. Moore	JMPC, 1078	Pirpirituba-PB	40	1.02	2A + 38F	1H, 4D
*T*. *elliptica* (DC.) Sandwith^*^	SAAL, 81	Santa Rita-PB	40	1.86	2A + 38F	1I, 4E
*T*. *rosea* (Bertol.) Bertero ex A. DC.	JMPC, 154	Areia-PB	40	1.51	2A + 2C + 36F	1J, 4F
*T*. *roseoalba* (Ridl.) Sandwith^*^	LPF, 14590	Campina Grande-PB	40	1.67	2A + 2C + 36F	1K, 4G
*Zeyheria tuberculosa* (Vell.) Bureau ex Verl.^*^	LPF, 14468	Maracás-BA	40	1.85	2A + 2C + 36F	1L, 4H
**Crescentieae**						
*Crescentia cujete* L.	JMPC, 137	Serra da Raiz-PB	40	1.21	2A + 38F	1A, 4I

* First chromosome count for the species.

** New cytotype for the species.

### Cytogenetic analyses

Mitosis was examined in root tips that had been pre-treated with 0.002 M 8-hydroxyquinoline (8-HQ) for 24 h at 4 ºC, fixed in 3:1 (v/v) absolute ethanol/glacial acetic acid for 30 min, and then stored in a freezer at -20 ºC. The roots were digested with an enzymatic solution (2% cellulase and 20% pectinase) for one hour at 37 ºC. Root tips were squashed in 45% acetic acid and coverslips were removed by freezing in liquid nitrogen. The samples were aged for three days at room temperature and stained with 10 μL of CMA (0.1 mg/mL) for 1 h, and then with 10 μL of DAPI (1 μg/mL) for 30 min. The samples were mounted in glycerol/McIlvaine’s buffer at pH 7.0 (1:1, v/v) and kept in the dark for three days (Cordeiro *et al.*, 2017).

The best metaphases were photographed using an AxioCam MRC5 digital camera and AxioVision 4.8 software (Carl Zeiss Microscopy GmbH, Jena Germany). Measurements were made using Uthscsa Image Tool (IT) v 3.0 software. The final images were prepared using Adobe Photoshop CS3 v 10.0 (Adobe Systems Incorporated, San Jose, USA). Chromosome morphology was determined using the centromeric index, following Guerra (1986).

### Base chromosome number and karyotype evolution

The base chromosome number analysis is based on 179 species of Bignoniaceae, distributed in all of the clades retrieved by Olmstead *et al.* (2009) for the family. The list of samples, and their chromosome numbers and respective references are presented in Table S1 (Supplementary Material). Karyotype and molecular phylogenetic data were compiled for representatives of the Bignoniaceae. The numbers of species analyzed in each Bignoniaceae clade and their chromosome numbers and frequencies are presented in a phylogeny adapted from Olmstead *et al.* (2009) to demonstrate their putative chromosome number evolution. Information concerning heterochromatin patterns is presented for Bignonieae, *Tabebuia* alliance, Tecomeae, and Jacarandeae. The chromosomes types (A, B, C, D, E and F) follow Cordeiro *et al.* (2017).

## Results

### Chromosome numbers

The chromosome number of 12 species of the *Tabebuia* alliance clade was analyzed, as well as those of three species of Jacarandeae, two species of Tecomeae, and two species of Bignonieae tribes. The karyotypes of the 19 species analyzed were predominantly symmetrical, principally with metacentric or sub-metacentric chromosomes. Their sizes ranged from 1.02 μm ± 0.13 in *Tabebuia aurea* (Silva Manso) Benth. & Hook. f. ex S. Moore to 2.19 μm ± 0.3 in *J. praetermissa*. The chromosome number of most of the *Tabebuia* alliance was 2*n* = 40 (*Crescentia* L., *Sparattosperma* Mart. ex Meisner, *Tabebuia* Gomez*,* and *Zeyheria* Mart.). However, *Handroanthus* Mattos showed 2*n* = 40 [*H. impetiginosus* (Mart. ex DC.) Mattos and *H. umbellatus*] as well as 2*n* = 80 [*H. chrysotrichus* (Mart. ex DC.) Mattos and *H. ochraceus* (Cham.) Mattos], and 2*n* = 120 [*H. serratifolius* (Vahl.) S.O.Grose]. The remaining species showed 2*n* = 36 [*Jacaranda mimosifolia* D.Don., *J. jasminoides* (Thunb.) Sandwith., *J. praetermissa*, and *Tecoma stans* (L.) Juss. ex Kunth], 2*n* = 38 [*Podranea ricasoliana* (Tafani) Sprague], or 2*n* = 40 (*A. citrinum* and *F. chica*) (Table 1).

New chromosome records are described for *Handroanthus umbellatus* (Sond.) Mattos, *Sparattosperma leucanthum* (Vell.) K.Schum, *Tabebuia elliptica* (DC.) Sandwith, *T. roseoalba* (Ridl.) Sand., and *Z. tuberculosa* (Vell.) Bureau ex Verl. (2*n* = 40; *Tabebuia* alliance), as well as for *Fridericia chica* (Bonpl.) L.G.Lohmann (2*n* = 40; Bignonieae tribe) and *Jacaranda praetermissa* Sandwith (2*n* = 36; Jacarandeae tribe). Additionally, a new cytotype is described for *Anemopaegma citrinum* Mart. ex DC. (2*n* = 40; Bignonieae tribe).

### Base chromosome number and karyotype evolution

**Figure 2:**
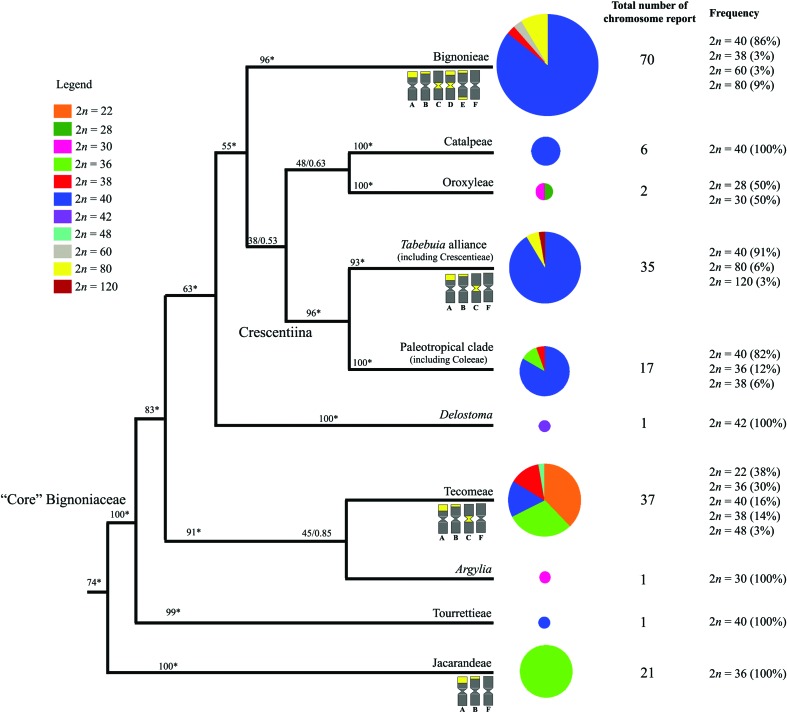
Chromosome numbers of the Bignoniaceae clades. Values on the branches indicate bootstrap parsimony analysis and the posterior probability of Bayesian inference; Asterisks indicate 100% posterior probabilities (topology and support values following Olmstead *et al.*, 2009). Circle sizes correspond to the numbers of species with chromosome records in each clade. Chromosomes types A, B, C, D, E and F follow Cordeiro *et al.* (2017).

The chromosome numbers of 179 species of Bignoniaceae (belonging to all of its clades) were compared (Table S1). Overall, most species showed 2n = 40 (67%) and 2*n* = 36 (19%). Chromosome numbers were compiled in a phylogeny adapted from Olmstead *et al.* (2009) to infer chromosome number evolution (Figure 2). The chromosome number 2*n* = 36 (*n* = 18) was principally distributed within the tribe Jacarandeae, while 2*n* = 40 (*n* = 20) appeared especially in the tribes Bignonieae and Catalpeae, in the clade Crescentiina, and in Tourrettieae. Other chromosome numbers occurred in *Argylia* (2*n* = 30) and *Delostoma* (2*n* = 42), and in the tribes Oroxyleae (2*n* = 28 and 30) and Tecomeae (2*n* = 22, 38 and 48).

### Heterochromatin patterns

**Figure 3:**
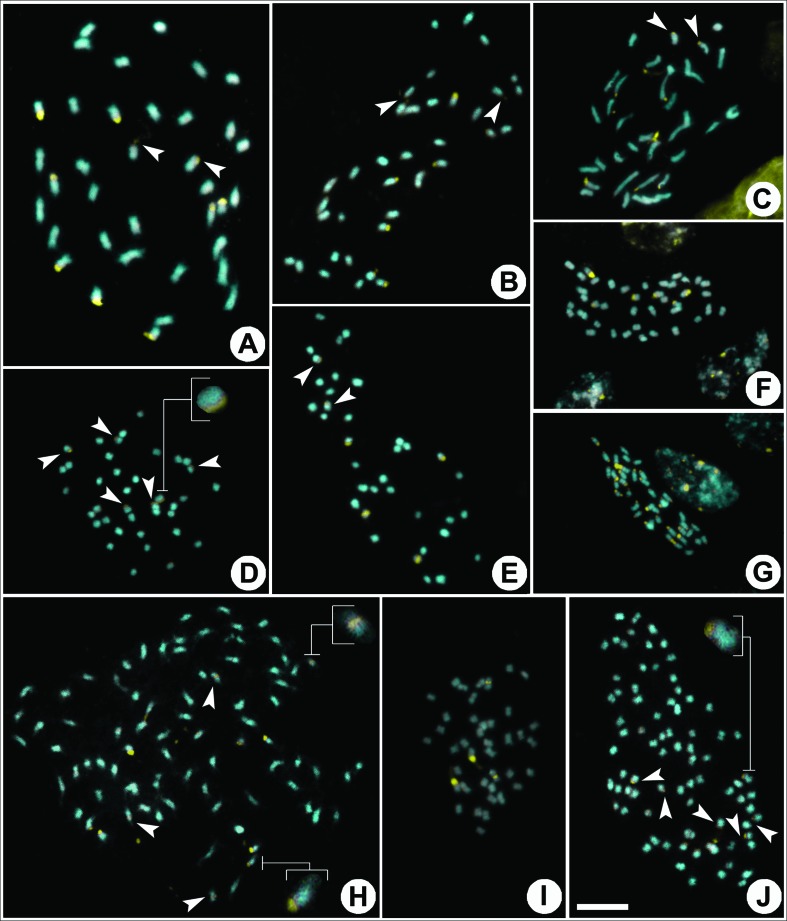
Distribution of heterochromatic bands (CMA^+^, in yellow) of species of Jaracandeae, Tecomeae, Bignonieae and the *Tabebuia* alliance. A. *Jacaranda jasminoides* (2*n* = 36), B. *J. mimosifolia* (2*n* = 36), C. *J. praetermissa* (2*n* = 36), D. *Podranea ricasoliana* (2*n* = 38), E. *Tecoma stans* (2*n* = 36), F. *Anemopaegma citrinum* (2*n* = 40), G. *Fridericia chica* (2*n* = 40), H. *Handroanthus chrysotrichus* (2*n* = 80), I. *H. impetiginosus* (2*n* = 40), J. *H. ochraceus* (2*n* = 80). Scale bar in J corresponds to 10 μm. Arrow heads indicate minor CMA bands; inserts in D, H and J highlight chromosomes with inconspicuous CMA bands.

**Figure 4:**
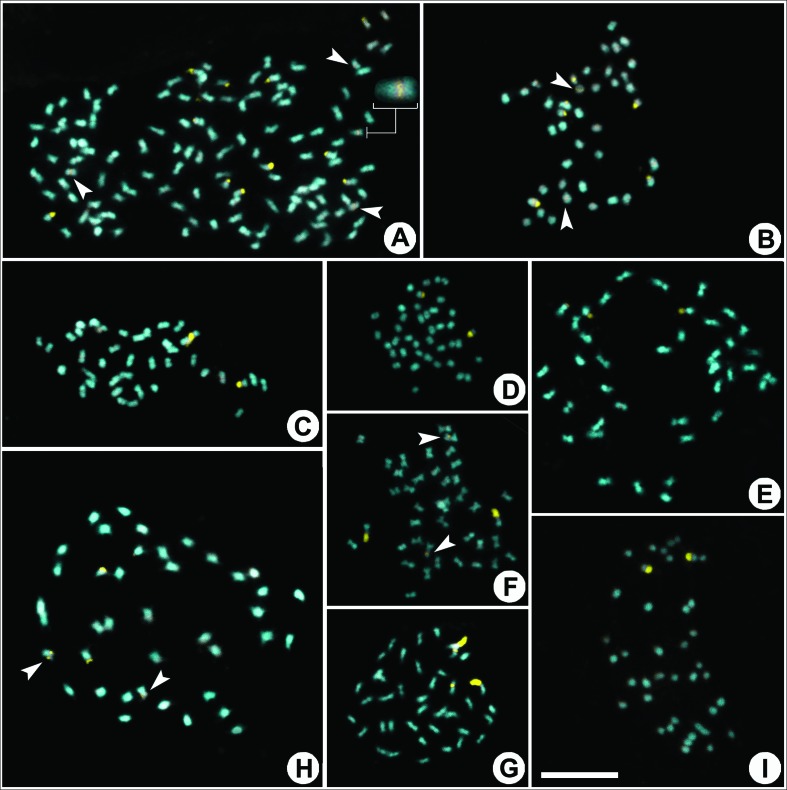
Distribution of heterochromatic bands (CMA^+^, in yellow) of species of the *Tabebuia* alliance (including Crescentieae). A. *Handroanthus serratifolius* (2*n* = 120), B. *H. umbellatus* (2*n* = 40), C. *Sparattosperma leucanthum* (2*n* = 40), D. *Tabebuia aurea* (2*n* = 40), E. *T. elliptica* (2*n* = 40), F. *T. rosea* (2*n* = 40), G. *T. roseoalba* (2*n* = 40), H. *Zeyheria tuberculosa* (2*n* = 40), I. *Crescentia cujete* (2*n* = 40). Scale bar in I corresponds to 10 μm. Arrow heads indicate minor CMA bands; inserts in A highlight chromosomes with inconspicuous CMA bands.

The heterochromatin banding patterns of the 19 species analyzed showed GC-rich (CMA^+^/DAPI^-^) bands located on the telomeric or proximal regions of the chromosomes (Figures 3 and 4). The species belonging to Jacarandeae, Tecomeae, and Bignonieae tribes had distinct patterns of CMA^+^/DAPI^-^ bands. Jacarandeae had five pairs of telomeric bands in *J. jasminoides* (Figure 3A) and *J. praetermissa* (Figure 3C), and four telomeric pairs in *J. mimosifolia* (Figure 3B). Tecomeae had three pairs of inconspicuous telomeric bands in *P. ricasoliana* (Figure 3D), and one telomeric pair plus two proximal pairs in *T. stans* (Figure 3E). Bignonieae displayed two telomeric pairs as well as two telomeric and proximal pairs in *A. citrinum* (Figure 3F), and 16 telomeric pairs and three telomeric pairs with bands on the short and long arm in *F. chica* (Figure 3G).

Most species in the *Tabebuia* alliance had karyotypes with a pair of chromosomes with large CMA^+^/DAPI^-^ telomeric bands, as seen in *Crescentia cujete* L. (Figure 4I), *S. leucanthum* (Figure 4C), *T. elliptica* (Figure 4E), and *T. aurea* (Figure 4D). Karyotypes with two telomeric and two proximal bands were observed in *H. impetiginosus* (Figure 3I), *Tabebuia rosea* (Bertol.) Bertero ex A.DC. (Figure 4F), *T. roseoalba* (Figure 4G), and *Z. tuberculosa* (Figure 4H). The remaining species of *Handroanthus* showed distinct heterochromatin patterns: four telomeric bands (two large and two small) and four proximal bands in *H. umbellatus* (Figure 4B), four small telomeric bands in *H. ochraceus* (Figure 3J), eight telomeric bands (four large and four small) and four proximal bands in *H. chrysotrichus* (Figure 3H), and ten telomeric bands (four large and six small) and four proximal bands in *H. serratifolius* (Figure 4A).

## Discussion

### Chromosome number evolution in Bignoniaceae

Raven (1975) suggested *x* = 7 as the ancestral base number for Bignoniaceae, with the most common *n* = 20 being generated by a six-fold polyploidization followed by the loss of one pair of chromosomes; that base number was suggested because he considered Oroxyleae (*n* = 14 and 15) to be the most primitive tribe in Bignoniaceae. Several cytological studies in Bignoniaceae (Goldblatt and Gentry, 1979; Piazzano, 1998; Chen *et al.*, 2004) agreed with the hypothesis of Raven (1975). More recent works, such as Piazzano *et al.* (2015), however, suggested *x* = 20 as the basic number of Bignoniaceae. The principal justification for that would be the large number of species with 2*n* = 40, and groups considered correlated with Bignoniaceae, such as Paulowniaceae and Schlegeliaceae, which also share the haploid number *n* = 20.

Molecular phylogeny, however, suggests a different story. Paulowniaceae and Schlegeliaceae are not closely related to Bignoniaceae (Olmstead *et al.*, 2009; Refulio-Rodriguez and Olmstead, 2014). According to Olmstead *et al.* (2009), the first diverging lineage within Bignoniaceae was Jacarandeae (2*n* = 36), followed by a strongly supported clade (core Bignoniaceae) with Tourrettieae (2*n* = 40), and then *Argylia* (2*n* = 30), Tecomeae (2*n* = 18, 22, 34, 36, 38, and 40), and a large clade including Oroxyleae (2*n* = 28, 30), Crescentiina (mostly 2*n* = 40, but also 36, 38, 80 and 120), and Bignonieae (mostly 2*n* = 40, but also 38, 60, and 80) (Figure 2). Among the most basal lineages (Jacarandeae, Tourrettieae, *Argylia*, Tecomeae, and *Delostoma*) only 8.7% of the species (five species) have 2*n* = 40, whereas 56.1% (32 species) show 2*n* = 36 (Table S1, Figure 2). Consequently, the haploid base number for the family is *x* ≠ 20. Very likely, the haploid number is *x* = 18, which was followed by an ascendant dysploidy (*n* = 18 → *n* = 20) in the most derived and diversified clades of the family.

Jacarandeae and Tourretieae are the most primitive group for Bignoniaceae. Jacarandeae include two genera (*Jacaranda* Juss. and *Digomphia* Benth.) and approximately 55 species that are widely distributed throughout the Neotropics (Gentry, 1980; Olmstead *et al.*, 2009). The chromosome number in the *Jacaranda* is very well characterized by the 2*n* = 36 (Cordeiro *et al.*, 2016b). Tourrettieae include two small genera subwoody to herbaceous vines (*Eccremocarpus* Ruiz & Pav. and *Tourrettia* DC.) and six species distributed in the Andes and north in the Central American Cordilleras to Mexico (Gentry, 1980; Olmstead *et al.*, 2009). There are chromosomal records in this tribe only for *Tourrettia lappacea* (L’Hér.) Willd. (2*n* = 40) (Goldblatt and Gentry, 1979). Although the chromosomal record for Tourrettieae and Jacarandeae are different, these two basal tribes share some traits, as the doubly compound leaves and pollen that is psilate and tricolpate (Olmstead *et al.*, 2009). Further sampling in Tourrettieae can confirm whether 2*n* = 40 is a typical chromosomal number for the tribe species or if there may be other chromosome numbers, as also observed in Tecomeae.

Tecomae is placed between the basal (Jacarandeae and Tourrettieae) and most derived clades of the Bignoniaceae (Crescentiina, Bignonieae, Catalpeae). The tribe is characterized by wide variations in chromosome numbers (2*n* = 22, 36, 38, 40, and 48), unlike other tribes where 2*n* = 36 (Jacarandeae) or 2*n* = 40 (Bignonieae, Catalpeae, and Crescentiina clade) predominate (Table S1, Figure 2). Variations in chromosome numbers in Tecomeae represent events of ascending and descending disploidy resulting in different chromosome numbers. The presence of *n* = 20 in Tourrettieae suggests that this number could have arisen at the Core Bignoniaceae by ascending disploidy, while the other numbers could have arisen by ascending (*n* = 21, 24) and descending (*n* = 11, 14, 15, 19) disploidy.

Most species of the derived clade comprising Catalpeae, Oroxyleae, Crescentiina, and Bignonieae (Olmstead *et al.*, 2009) have the karyotype 2*n* = 40. Among the 122 species with known chromosome numbers within this clade, 92.6% (113 species) show 2*n* = 40. Only six species show 2*n* ≠ 40 [two species of *Mansoa* DC. in Bignonieae, two species of Oroxyleae, and *Spathodea campanulata* P. Beauv. and *Radermacheraxylocarpa* (Roxb.) Roxb. ex K. Schum.; Table S1]. The remaining species are polyploids of the haplotype *n* = 20 (2*n* = 60, 80, 120). This large clade comprises around 80% of the species of Bignoniaceae (Olmstead *et al.*, 2009), which makes 2*n* = 40 the most common karyotype in the family. The four tribes and informal groups in this derived clade show marked geographical patterns. The most species-rich tribe (Bignonieae) is Neotropical (Fischer *et al.*, 2004) as is one lineage of the informal Crescetiina (which also has one Paleotropical clade) (Grose and Olmstead, 2007). Catalpeae is from temperate North America and China and the tropical Greater Antilles (Gentry, 1980; Olsen and Kirkbride Jr, 2017), while the smallest tribe, Oroxyleae, is from tropical southern and southeastern Asia and Malaysia (Olmstead, 2013). Their wide distribution around the world and the high numbers of species in those tribes make *n* = 20 the most common haploid number in Bignoniaceae. The haploid number *n* = 20 could be related to actual diversity and the occupation of a wide variety of habitats.

Reported chromosome numbers suggest that polyploidy is restricted to the clades *Tabebuia* alliance and Bignonieae (Goldblatt and Gentry, 1979; Piazzano, 1998; Firetti-Leggieri *et al.*, 2011, 2013; Cordeiro *et al.*, 2017). Reproductive analyses of *Handroanthus* and *Anemopaegma* Mart. ex Meisn. indicated self-pollination, sporophytic and pseudogamous apomixis, and polyembryonic seeds (Piazzano, 1998; Bittencourt Jr and Moraes, 2010; Firetti-Leggierri *et al.*, 2013) – which are common features in polyploid species (Piazzano, 1998; Firetti-Leggieri *et al.*, 2013). Piazzano *et al.* (2015) suggested that the polyploidy observed in those species probably originated by meiotic alteration, leading to the production of non-reduced gametes. The absence of a morphological continuum between sympatric species of the same genera (personal observations) suggests an autopolyploid origin.

### Heterochromatin patterns

Heterochromatin in the basal lineage of Jacarandeae (Bignoniaceae) is composed exclusively by 8-16 terminal CMA^+^ bands, while the following lineages (Tecomeae, Bignonieae, *Tabebuia* alliance) also demonstrate pericentromeric CMA^+^ bands, but with reductions in the numbers of terminal CMA^+^ blocks (Cordeiro *et al.*, 2016b, 2017; Figures 3 and 4). In certain plant groups, such as the Caesalpinia group (Van-Lume *et al.*, 2017), and sect. *Acanthophora* of *Solanum* L. (Chiarini *et al.*, 2014) and *Nierembergia* Ruiz & Pav. (Acosta *et al.*, 2016), the heterochromatin patterns appear to follow a specific evolutionary pattern for the species in the different clades. In genera such as *Lycium* L. (Stiefkens *et al.*, 2010), *Pereskia* Mill. (Castro *et al.*, 2016), and *Ceiba* Mill. (Figueredo *et al.*, 2016), however, the heterochromatin pattern appears to be quite conserved and demonstrate only small variability among the different species. In most plant groups, however, heterochromatin patterns tend to be random and quite distinct, even among closely related species (Berjano *et al.*, 2009; Scaldaferro *et al.*, 2012; Grabiele *et al.*, 2018; Van-Lume and Souza, 2018). For Bignoniaceae as a whole, three heterochromatin patterns can be seen, with the occurrence of a specific pattern for Jacarandeae (terminal CMA^+^ blocks), conserved patterns for the diploid species of the *Tabebuia* alliance (two terminal CMA^+^ blocks and 0-2 pericentromeric CMA^+^ blocks), and a pattern of random attributions in relation to the numbers and positions of CMA^+^ blocks in the tribe Binonieae (Cordeiro *et al.*, 2017).

The heterochromatin banding patterns of the Bignonieae tribe in Bignoniaceae (Cordeiro *et al.*, 2017) are characterized by strong differences in the sizes and locations of the CMA^+^ blocks, and six chromosome types are recognized based on heterochromatic regions. The two species of Bignonieae analyzed here confirm the patterns described before for the tribe, with the occurrence of type A (large telomeric CMA^+^ bands), type B (small telomeric CMA^+^ bands), type D (telomeric and proximal CMA^+^ bands), and type F chromosomes (showing a lack of heterochromatic bands) in *A. citrinum*, and type A, B, E (two telomeric CMA^+^ bands) and F chromosomes in *F. chica* (Table 1, Figure 2). The species sampled in *Tabebuia* alliance, Jacarandeae, and Tecomeae have four chromosome types (according to Cordeiro *et al.*, 2017): type A, type B, type C (proximal CMA^+^ bands), and type F.

The pattern of two CMA^+^ telomeric bands (chromosome type A) seen in most species of the *Tabebuia* alliance is very common among Angiosperms, and usually corresponds to a nucleolar organizer region (Guerra, 2000; Roa and Guerra, 2012). Telomeric CMA bands are most likely related to rDNA sites as seen in most plant species (Barros e Silva *et al.*, 2010; Castro *et al.*, 2016; Marinho *et al.*, 2018). Differences among species could be related tochromosome rearrangements and the amplification and reduction of rDNA sites caused by satellites or transposable sequences (Mehrotra and Goyal, 2014; Evtushenko *et al.*, 2016; Saze, 2018).

The vegetative morphologies of *Handroanthus* species having yellow corollas are very similar (Gentry, 1992), with *H. chrysotrichus* and *H. ochraceus* being very close, even when comparing their flowers, leaves, and fruits. Those two species show a continuum of morphological variations, and hybridization or introgression has therefore been suggested (Gentry, 1992; Bittencourt Jr and Moraes, 2010). However these species have a distinctive heterochromatin banding pattern 4A + 4B + 4C in *H. chrysotrichus* and 4B in *H. ochraceus*. Similarly, *T. roseoalba* and *T. elliptica* have very similar flowers and fruits, although they can be differentiated by their 3- or 5-foliolate leaves respectively (Gentry, 1992). The heterochromatin banding patterns of those two species are distinct, with the former having two proximal plus two telomeric bands (2A + 2C), while the latter has only two telomeric bands (2A). While banding patterns are still seldom-used in taxonomic studies, the results reported here support their utility in such analyses.

The chromosome numbers and heterochromatin banding patterns of *J. jasminoides*, *J. praetermissa* and *J. mimosifolia* support published data for the genus (Cordeiro *et al.*, 2016b). *Jacaranda* is one of the largest genera of Bignoniaceae, with more than 50 species widely distributed in the Neotropics (Gentry and Morawetz, 1992). The genus is very well characterized by the chromosome number 2*n* = 36 (Morawetz, 1982; Cordeiro *et al.*, 2016b) and by having 8 to 16 small and terminal CMA^+^ bands (Cordeiro *et al.*, 2016b). In addition to its stable chromosome features, *Jacaranda* has a very consistent morphology, with all of its species having pinnate or bipinnate leaves, calyx lobes that are deeply divided, staminodes longer than the stamens, and oblong and strongly flattened capsules opening through a rupture perpendicular to the septum (Morawetz, 1982; Gentry and Morawetz, 1992; Olmstead *et al.*, 2009).

The heterochromatin banding patterns of Tecomeae have been poorly studied. The karyotypes of the two species of the tribe analyzed here, however, were relatively distinct from the species belonging to Jacarandeae, the *Tabebuia* alliance clades, and Bignonieae species (Cordeiro *et al.*, 2017). Although *T. stans* shows 2*n* = 36, its heterochromatin banding pattern (2A + 4C) is quite distinct from species with similar chromosome numbers, such as *Jacaranda* (8-16 A + B; Cordeiro *et al.*, 2016b). Regarding *P. ricasoliana*, this species has an uncommon chromosome number for the Bignoniaceae (2*n* = 38) and six small terminal CMA^+^ bands – a unique pattern in the family (which usually has at least two large terminal CMA^+^ bands) (Cordeiro *et al.*, 2016b, 2017). Although there is still little data available concerning banding patterns in Tecomeae, their lack of synapomorphies (Olmstead *et al.*, 2009), wide distributions (Olmstead, 2013), high variability of life forms (including herbs, shrubs, trees, and lianas), environments occupied (from tropical to temperate forests, to both Andean and Himalayan mountains), and variations in chromosome numbers of the species within this clade, make this tribe one of the major challenges in Bignoniaceae.

## Conclusion

The revision of the chromosome numbers previously reported for Bignoniaceae, allied to previous phylogenetic studies for the family, support a basic haploid chromosome number different from 20 for the family. The most likely primary base number for the family is *x* = 18, which is the most common haploid number among its basal lineages. Ascending disploidy leading to *x* = 20 is consistent with the chromosome numbers found in the most derived and diversified lineages, where that number predominates. A broad study involving reconstructions of chromosome counts in families related to Bignoniaceae, as well as in all of its clades, would help clarify the evolution of the karyotype of the family.

The chromosomes of the *Tabebuia* alliance showed only GC-rich bands (CMA^+^/DAPI^-^) located in telomeric or proximal regions. The banding pattern within that clade was more variable than seen in *Jacaranda,* but less variable than in Bignonieae. Despite the intermediate level of variation observed, heterochromatin banding patterns offer a promising tool for distinguishing species, especially in the morphologically complex genus *Handroanthus*.

## Supplementary material

The following online material is available for this article:

Table S1 Chromosome numbers recorded for the Bignoniaceae family and their respective bibliographic references.
