# Supplementary material for: Heterochromatin and numeric chromosome evolution in Bignoniaceae, with emphasis on the Neotropical clade Tabebuia alliance
Source: Genet Mol Biol. 2020 Feb 27;43(1):e20180171. doi: 10.1590/1678-4685-GMB-2018-0171 (PMC7229889; doi:10.1590/1678-4685-GMB-2018-0171)
Supplement: Supplementary file 1 [file 1415-4757-GMB-43-1-e20180171-s001.pdf]

Supplementary Material to “Heterochromatin and numeric chromosome evolution in Bignoniaceae, with emphasis on the Neotropical clade *Tabebuia alliance*”

Table S1 - Chromosome numbers recorded for the Bignoniaceae family and their respective bibliographic references.

Taxa*	2*n*	Source
**Jacarandeae**		
Jacaranda acutifolia Bonpl.	36	R15
J. bracteata Bureau & K. Schum.	36	CO16b
J. brasiliana (Lam.) Pers.	36	MWZ82, CO16b
J. caerulea (L.) J.St.-Hil.	36	MWZ82
J. caroba (Vell.) DC.	36	FM00
J. cuspidifolia Mart.	36	CO16b
J. decurrens Cham.	36	GG79
J. hesperia Dugand	36	GG79, MWZ82
J. irwinii A.H. Gentry	36	CO16b
J. jasminoides (Thunb.) Sandwith	36	CO16b, Present study
J. macrantha Cham.	36	MWZ82
J. micrantha Cham.	36	MWZ82
J. mimosifolia D. Don	36	GG79, P98, CO16b, Present study
J. paucifoliolata Mart. ex DC.	36	DSun
J. praetermissa Sandwith	36	Present study
J. puberula Cham.	36	MWZ82
J. pulcherrima Morawetz,	36	MWZ82
J. rufa Silva Manso	36	MWZ82
J. rugosa A.H. Gentry	36	CO16b
J. subalpina Morawetz	36	MWZ82
J. ulei Bureau & K.Schum.	36	DSun
**Tourretieae**		
Tourrettia lappacea (L'Hér.) Willd.	40	GG79

Taxa*	2*n*	Source
Argylia		
Argylia uspallatensis DC.	30	GG79
**Tecomeae**		
Campsis grandiflora (Thunb.) K.Schum.	36, 38, 40	V44, MO74, GG79
C. radicans (L.) Seem.	40	V44, MO74, GG79
Campsis × *tagliabuana* (Vis.) Rehder	40	V44, GG79
Fernandoa adenophylla (Wall. ex G. Don) Steenis	40	MO74, GJ90
Incarvillea beresowskii Batalin	22	CH04
I. compacta Maxim.	22	MO74, CH04
I. delavayi Bureau & Franch.	22	CH04
I. diffusa Royle	22	CH04, KS12
I. dissectifoliola Q. S. Zhao	22	CH04
I. emodi (Royle ex Lindl.)	22	KS12
I. forrestii Fletcher	22	X02
I. lutea Bureau & Franch.	22	CH04
I. mairei (H. Lév.) Grierson	22, 36	MO74, CH04
I. olgae Regel	22	MO74, CH04
I. semiretschenskia (B.Fedtsch.) Grierson	22	GG79
I. sinensis Lam.	22	MO74, CH04
I. younghusbandii Sprague	22	CH04
I. zhongdianensis Grey-Wilson	22	CH04
Lamiodendron magnificum Steenis	48	OK98
Pajanelia longifolia (Willd.) K.Schum.	40	V44
Pandorea jasminoides (Lindl.) K.Schum.	38	MO74, GG79
P. pandorana Steenis	38	V44
Podranea ricasoliana (Tafani)	38	P15, Present study

**Taxa***	**2*n***	**Source**
Sprague		
Tecoma capensis (Thunb.) Lindl.	36	GG79, P98
T. fulva subsp. *arequipensis* (Sprague) J.R.I.Wood	36	GG79
T. fulva subsp. *garrocha* (Hieron.) J. R. I. Wood	36	P98, GG79
T. sambucifolia Kunth	36	GG79
T. stans (L.) Juss. ex Kunth	40, 36	V44, GG79, P98, P15, Present study
T. stans var. *angustatum* Rehder	36	R15
T. stans var. *velutina* DC.	36	GG79
Tecomanthe dendrophila (Blume) K.Schum.	36	GG79
T. speciosa W.R.B.Oliv.	38	GG79
Tecomaria capensis (Thunb.) Lindl.	36	GG79
Delostoma		
Delostoma lobbii Seem.	42	GG79
**Bignonieae**		
Adenocalymma campicola (Pilg.) L.G.Lohmann	40	DSun
A. cidii (A.H.Gentry) L.G.Lohmann	40	R15
A. divaricatum Miers	40	CO17
A. imperatoris-maximilianii (Wawra) L.G.Lohmann	40	CO16a, CO17
A. marginatum (Cham.) DC.	40	P98, P15
A. patulum (Miers) L.G.Lohmann	40	GG79
A. pedunculatum (Vell.) L.G.Lohmann	40	DSun
A. peregrinum (Miers) L.G.Lohmann	40	DSun
Amphilophium bauhinioides (Bureau ex Baill.) L.G.Lohmann	40	CO16a, CO17
A. buccinatorium (DC.) L. G.	40	MO74, GG79

Taxa*	2*n*	Source
Lohmann		
A. crucigerum (L.) L.G.Lohmann	40	GG79, P98, P15, CO17
A. cynanchoides (DC.) L.G.Lohmann	40	P98, P15
A. elongatum (Vahl) L.G.Lohmann	40	CO16a, CO17
A. paniculatum (L.) Kunth	40	GG79, P98, P15
A. pannosum (DC.) Bureau & K. Schum.	40	P98
A. scabriusculum (Mart. ex DC.) L.G.Lohmann	40	CO17
Anemopaegma acutifolium DC.	80	FL11
A. album Mart. ex DC.	40	FL11, CO17
A. arvense (Vell.) Stellfeld ex De Souza	80	FL11
A. chamberlaynii (Sims) Bureau & K.Schum.	40	MO74, GG79, FL11
A. citrinum Mart. ex DC.	40, 60	CO17, Present study
A. glaucum Mart. ex DC.	80	FL11
A. laeve DC.	40	CO16a, CO17
A. orbiculatum (Jacq.) DC.	40	FL11
A. scabriusculum Mart. ex DC.	80	FL11
Bignonia aequinoctialis L.	40	GG79, GJ90, A12
B. binata Thunb.	40	GG79, P98, P15
B. callistegioides Cham.	40	P98
B. capreolata L.	40	MO74, GG79
B. corymbosa (Vent.) L.G.Lohmann	40	GG79, SIM54
B. diversifolia Kunth	40	MO74, GG79
B. magnifica W. Bull	40	MO74, GG79
Callichlamys latifolia (Rich.) K. Schum.	40	GG79, SIM54
Cuspidaria bracteata (Baill. ex Bureau & K.Schum.) L.G.Lohmann	40	CO16a

Taxa*	2*n*	Source
C. cinerea (Bureau ex K.Schum.) L.G.Lohmann	40	GG79
C. convoluta (Vell.) A. Gentry	40	P98, P15
C. lateriflora (Mart.) DC.	40	CO17
C. sceptrum (Cham.) L.G.Lohmann	40	DSun
Dolichandra cynanchoides Cham.	40	GJ90, P98, P15
D. dentata (K.Schum.) L. G. Lohmann	40	P98, P15
D. quadrivalvis (Jacq.) L.G.Lohmann	40	CO16a, CO17
D. uncata (Andrews) L. G. Lohmann	40	P98
D. unguis-cati (L.) L. G. Lohmann	40, 80	GG79, P98, P15, CO16a, CO17
Fridericia chica (Bonpl.) L.G.Lohmann	40	Present study
Fridericia conjugata (Vell.) L.G.Lohmann	40	CO16a
F. erubescens (DC.) L.G.Lohmann	40	CO16a, CO17
F. limae (A.H.Gentry) L.G.Lohmann	40	CO17
F. platyphylla (Cham.) L.G.Lohmann	40	CO16a
F. pubescens (L.) L.G.Lohmann	40	CO16a, CO17
F. subverticillata (Bureau & K. Schum.) L.G. Lohmann	40	CO17
Lundia corymbifera (Vahl) Sandwith	40	GG79
L. longa (Vell.) DC.	40	CO16a, CO17
Mansoa difficilis (Cham.) Bureau & K.Schum.	38	CO16a, CO17
M. hymenaea (DC.) A.H.Gentry	38	GG79
Martinella obovata (Kunth) Bureau & K.Schum.	40	GG79
Pleonotoma jasminifolia (Kunth) Miers	40	GG79

Taxa*	2*n*	Source
Pyrostegia venusta (Ker Gawl.) Miers	40, 60, 80	JH56, GG79, P98, CO16a, CO17
Stizophyllum perforatum (Cham.) Miers	40	GG79
S. riparium (Kunth) Sandwith	40	GG79, CO16a
Tanaecium dichotomum (Jacq.) Kaehler & L.G.Lohmann	40	GG79, P98, CO16a, CO17
T. jaroba Sw.	40	MO74, GG79
T. pyramidatum (Rich.) L. Lohmann	40	GG79, SIM54
T. selloi (Spreng.) L. G. Lohmann	40	P98, CO17
Tynanthus micranthus Corr. Méllo ex K. Schum.	40	P98, P15
Xylophragma myrianthum (Cham. ex Steud.) Sprague	40	CO17
X. seemannianum (Kuntze) Sandwith	40	GG79
**Catalpeae**		
Catalpa bignonioides Walter	40	MO74, GG79, P15
C. bungei C.A.Mey.	40	V44
C. ovata G. Don	40	MO74, GG79, GJ90
C. speciosa Teas	40	MO74, GG79
Catalpa × *erubescens* Carrière	40	MO74, GG79
Chilopsis lineares (Cav.) Sweet.	40	MO74, GG79
**Oroxyleae**		
Millingtonia hortensis L. f.	30	GG79, GJ90
Oroxylum indicum (L.) Kurz	28	GG79, GJ90
**Crescentiina, Paleotropical clade**		
Dolichandrone falcata (Wall. ex DC.) Seem.	40	GJ90
D. spathacea (L.f.) Seem.	40	MO74, GG79
D. stipulata (Wall.) C.B. Clarke	40	GG79
Fernandoa adenophylla (Wall. ex G.Don)	40	GG79

**Taxa***	**2*n***	**Source**
Kigelia africana (Lam.) Benth.	40	MO74, GJ90
Markhamia lutea (Benth.) K. Schum.	40	MO74, GG79, GJ90
M. platycalyx (Baker) Sprague	40	GG79
M. stipulata (Wall.) Seem	40	MO74
Newbouldia laevis (P.Beauv.) Seem.	40 (38)	GG79
Pajanelia longifolia (Willd.) K.Schum.	40	V44, GG79
Radermachera xylocarpa (Roxb.) Roxb. ex K. Schum.	36, 40	GG79, GJ90
Spathodea campanulata P. Beauv.	36, 38	GG79
Stereospermum chelonoides (L.f.) DC.	40	GG79, GJ90
S. kunthianum Cham.	40	GG79
S. tetragonum DC.	40	R15
**Coleeae**		
Phyllarthron comorense DC.	40	V44
**Crescentiina, *Tabebuia* alliance clade**		
Cybistax antisyphilitica (Mart.) Mart.	40	OR08
Handroanthus capitatus (Bureau & K.Schum.) Mattos	40	A02, AN07
H. chrysanthus subsp. *chrysanthus* (Jacq.) Grose	40	A02, AN07
H. chrysanthus subsp. *meridionalis* (A. H. Gentry) S. O. Grose	40	GG79, GJ90, P98
H. chrysotrichus (Mart. ex DC.) Mattos	40, 80	P98, P15, Present study
H. guayacan (Seem.) S.O.Grose	40	V44, GG79
H. heptaphyllus Mattos	40	GG79, P98, P15

**Taxa***	**2*n***	**Source**
H. impetiginosus Mattos	40	GG79, P98, P15, Present study
H. ochraceus subsp. *ochraceus* (Cham.) Mattos	40, 80	P15, Present study
H. ochraceus subsp. *heterotrichus* (DC.) S.O.Grose	40	AN07
H. ochraceus subsp. *neochrysanthus* (A.H.Gentry) S.O.Grose	40	AN07
H. pulcherrimus (Sandwith) Mattos	40	P98, P15
H. serratifolius (A. H. Gentry) S.Grose	40, 120	SIM54, GG79, A13, Present study
H. umbellatus (Sond.) Mattos	40	Present study
Roseodendron donnell-smithii (Rose) Miranda	40	SC78
Sparattosperma leucanthum (Vell.) K.Schum.	40	Present study
Tabebuia aurea (Silva Manso) Benth. & Hook.f. ex S.Moore	40	P15, Present study
T. elliptica (DC.) Sandwith	40	Present study
T. heterophylla (DC.) Britton	40	V44, GG79
T. nodosa (Griseb.) Griseb.	40	GG79
T. pallida (Lindl.) Miers	40	SC96, SIM54
T. rosea (Bertol.) Bertero ex A. DC.	40	GG79, SC96, Present study
T. roseoalba (Ridl.) Sand.	40	Present study
Zeyheria montana Mart.	40	DSun
Z. tuberculosa (Vell.) Bureau ex Verl.	40	Present study
**Crescentieae**		
Amphitecna latifolia (Mill.) A.H.Gentry	40	MO74, SIM54
A. montana L.O.Williams	40	MO74, SIM54
Crescentia cujete L.	40	MO74, SIM54, Present study
Parmentiera aculeata (Kunth) Seem.	40	V44

Taxa*	2*n*	Source
P. cereifera Seem.	40	V44, SIM54
P. macrophylla Standl.	40	GG79
P. valerii Standl.	40	G89

*Synonyms corrected following Lohmman and Ulloa (2016).
Source: A02 - Alcorcés de Guerra (2002); A12 - Alcorcés de Guerra (2012); A13 - Alves *et al*. (2013); AN07 - Alcorcés de Guerra and Natera (2007); CH04 - Chen *et al*. (2004); CO16a - Cordeiro *et al*. (2016a); CO16b - Cordeiro *et al*. (2016b); CO17 - Cordeiro *et al*. (2017); DSun - Diana S. Sampaio (unpublished data); FL11 - Firetti-Leggieri *et al*. (2011); FM00 - Forni-Martins and Martins (2000); G89 - Goldblatt (1989); GG79 - Goldblatt and Gentry (1979); GJ90 - Goldblatt and Johnson (1990); JH56 - Joshi and Hardas (1956); KS12 - Kaur and Singhal (2012); MO74 - Moore (1974); MWZ82 - Morawetz (1982); OK98 - Oginuma *et al*. (1998); OR08 - Ortolani *et al*. (2008); P98 - Piazzano (1998); P15 - Piazzano *et al*. (2015); R15 - Rice *et al*. (2015); SC78 - Sarkar *et al*. (1978); SC96 - Soontornchainaksang and Chaiyasut (1996); SIM54 - Simmons (1954); V44 - Venkatasubban (1944); X02 - Xiao *et al*. (2002).

Reference Supplementary Material

Alcorcés de Guerra N (2002) Cariología de dos especies del gênero *Tabebuia* Gomes (Bignoniaceae). UDO Agrícola 2: 4-21.

Alcorcés de Guerra N (2012) Meiosis en *Cydista aequinoctialis* (L.) Miers. (Bignoniaceae). UDO Agrícola 12: 69-73.

Alcorcés de Guerra N and Natera JRM (2007) Chromosome numbers of three *Tabebuia* species (Bignoiaceae). Nord J Bot 25: 359-360.

Alves MF, Duarte MO, Oliveira PE and Sampaio DS (2013) Self-sterility in the hexaploid *Handroanthus serratifolius* (Bignoniaceae), the national flower of Brazil. Acta Bot Brasilica 27: 714-722.

Chen ST, Zhou ZK, Guan KY and Nakata M (2004) Karyomorphology of *Incarvillea* (Bignoniaceae) and its implications in distribution and taxonomy. Bot J Linn Soc 144: 113-121.

Cordeiro JMP, Almeida EM, Lima SAA, Assis FNM, Souza LGR and Felix LP (2016a) In: Marhold K and Kučera J (Eds). IAPT/IOPB chromosome data 23. Taxon 65: 1455-1458.

Cordeiro JMP, Lima SAA, Paz SN, Santos MAS and Felix LP (2016b) Karyotype evolution in the genus *Jacaranda* Juss. (Jacarandeae, Bignoniaceae): chromosome numbers and heterocromatin. Genet Mol Res 15: gmr15048973.

Cordeiro JMP, Kaehler M, Souza G and Felix LP (2017) Karyotype analysis in Bignonieae (Bignoniaceae): chromosome numbers and heterochromatin. An Acad Bras Cienc 89: 2697-2706.

Firetti-Leggieri F, Costa IR, Lohmann LG, Semir J and Martins ERF (2011) Chromosome studies in Bignonieae (Bignoniaceae): The first record of polyploidy in *Anemopaegma*. Cytologia 76: 185-191.

Forni-Martins E and Martins FR (2000) Chromosome studies on Brazilian cerrado plants. Genet Mol Biol 23: 947-955.

Goldblatt P (1989) Miscellaneous chromosome counts in Asteraceae, Bignoniaceae, Proteaceae, and Fabaceae. Ann Missouri Bot Gard 76: 1186-1188.

Goldblatt P and Gentry AH (1979) Cytology of Bignoniaceae. Bot Not 132: 475-482.

Goldblatt P and Johnson DE (1990) Index to plant chromosome numbers 1986-1987. Monogr Syst Bot 30: 1-243.

Joshi AB and Hardas MW (1956) Ploidy in two bignoniaceous garden climbers. Indian J Genet Plant Breed 16: 57-59.

Kaur D and Singhal VK (2012) In: Marhold K and Breitwieser I (Eds). IAPT/IOPB chromosome data 13. Taxon 61: 894-895.

Lohmann LG and Ulloa CU (2016) Bignoniaceae. In: iplants prototype Checklist. www.iplants.org (January 14, 2018).

Moore RJ (org) (1974) Index to plant chromosome numbers for 1972. Regnum Veg 91: 1-108.

Morawetz W (1982) Morphologisch-ökologische differenzierung, biologie, systematic und evolution der Neotropischen gattung *Jacaranda* (Bignoniaceae). Denkschriften der Österreichischen Akademie der Wissenschaften. Springerverlag, Wien.

Oginuma K, Kiaptranis R, Damas K and Tobe H (1998) A cytological study of some plants from Papua New Guinea. Acta Phytotaxon Geobot 49: 105-114.

Ortolani FA, Mataqueiro MF, Moro JR, Moro FV and Damião Filho CF (2008) Morfo-anatomia de plântulas e número cromossômico de *Cybistax antisyphilitica* (Mart.) Mart. (Bignoniaceae). Acta Bot Brasilica 22: 345-353.

Piazzano M (1998) Números cromosómicos em Bignoniaceae de Argentina. Kurtziana 26: 179-219.

Piazzano M, Las Peñas ML, Chiarini F and Bernardello G (2015) Karyotypes and DNA content in Bignoniaceae. Caryologia 68: 175-183, 2015.

Rice A, Glick L, Abadi S, Einhorn M, Kopelman NM, Salman-Minkov A, Mayzel J, Chay O and Mayrose I (2015) The Chromosome Counts Database (CCDB) – a community resourse of plant chromosome numbers. New Phytol 206: 19-26.

Sarkar AK, Chakraborty M, Saha NC, Das SK and Hazra D (1978) IOPB chromosome number reports LXII. Taxon 27: 519-535.

Simmonds NW (1954) Chromosome behavior in some tropical plants. Heredity 8: 139-146.

Soontornchainaksang P and Chaiyasut K (1996) Cytogenetic study of some thai species of flowering plants. Thai For Bull (bot) 24: 50-61.

Venkatasubban KR (1944) Cytological studies in Bignoniaceae. Part IV. The cytology of *Dolichandrone rheedii* Seem. and allied genera. Proc Ind Acad Science B 21: 77-92.

Xiao H, Zhou Q, Gu Z and Guan K (2002) Karyomorphology of six *Incarvillea* species. Acta Bot Yunnanica 24: 87-93.
